# Effectiveness of a comprehensive integrated module using interactive lectures and workshops in understanding and knowledge retention about infant feeding practice in fifth year medical students: a quasi-experimental study

**DOI:** 10.1186/s12909-016-0705-2

**Published:** 2016-08-18

**Authors:** Damayanti Rusli Sjarif, Klara Yuliarti, Luh Karunia Wahyuni, Tjhin Wiguna, Titis Prawitasari, Yoga Devaera, Henni Wahyu Triyuniati, Andika Afriansyah

**Affiliations:** 1 Division of Nutrition and Metabolic Disease, Department of Pediatrics, Cipto Mangukusumo Hospital / Faculty of Medicine, Universitas Indonesia, Jl. Diponegoro 71, Jakarta, 10430 Indonesia; 2 Department of Physical Medicine and Rehabilitation, Faculty of Medicine, Universitas Indonesia, Cipto Mangukusumo Hospital, Jakarta, 10430 Indonesia; 3 Department of Psychiatry, Faculty of Medicine, Universitas Indonesia, Cipto Mangunkusumo Hospital, Jakarta, 10430 Indonesia

**Keywords:** Infant feeding practice, Comprehensive integrated module, Nutrition, Medical student

## Abstract

**Background:**

Sixty percent of the 10.9 million under-5 deaths every year are related to malnutrition. More than two thirds of malnutrition is caused by inappropriate infant feeding practice. Only 35 % of mothers worldwide provide 4 months of exclusive breast-feeding, while complementary feeding is often untimely, nutritionally inadequate, hygienically poor, and improperly delivered. The existing pediatric nutrition module in our institution does not include proper delivery of food that involves oral–motor skills and feeding behavior. To scale up the knowledge and skill of medical students regarding evidence-based infant feeding practice, we designed a new module composed of comprehensive and integrated lectures with additional multidisciplinary lectures on oral–motor skill development and feeding behavior.

**Methods:**

A quasi-experimental study was conducted to evaluate the efficacy of the new module compared to the previous module. Fifth year medical students of Universitas Indonesia were divided into intervention and control groups. The control group received lectures and a paper-based workshop. The intervention group received comprehensive and integrated interactive lectures with additional multidisciplinary lectures on oral–motor skill development and behavioral approaches to feeding problems. A hands-on workshop using real cases shown on recorded video and role-play sessions was also presented to the intervention group. A pre-/post-test, 3-month retention test, and Observed Structured Clinical Examination (OSCE) were performed to evaluate understanding, knowledge retention, and counseling skills.

**Results:**

A linear mixed effect model with a random intercept analysis for pre-test, post-test, and retention test scores showed significant higher result for intervention group compared to control group (*p* < 0.001). Comprehensive knowledge and counselling skills were better in the intervention group than in the control group as shown by the OSCE score (68.6 vs 59.3, *p* < 0.001).

**Conclusions:**

Our comprehensive integrated infant feeding practice module, which incorporates multidisciplinary learning processes and an interactive hands-on workshop with a role-play session yields better knowledge understanding and counseling skills compared with the existing module. Comprehensive knowledge and good counseling skills of medical students as future doctors are a pre-requisite to provide effective education to parents to support successful infant feeding practices.

## Background

Sixty percent of the 10.9 million under-5 deaths every year are related to malnutrition [1]. Poor breastfeeding and complementary feeding practices, coupled with high rates of infectious diseases, are the principal proximate causes of malnutrition during the first two years of life [1]. For this reason, the most effective intervention to reduce under-5 mortality and morbidity rates in developing countries is implementing appropriate infant feeding practices [1, 2].

Successful infant feeding is influenced by several factors, such as the quality and quantity of the food, development of oral–motor skills, and establishment of good rapport between the caregiver and infant during feeding [3–5]. A study in Pakistan showed that the mothers’ knowledge regarding complementary feeding was low [6]. In this previous study, 21 % of infants were aged 2–3 months and had received complementary feeding, while 19 % of infants were aged 6–8 months and were still on exclusive breastfeeding without receiving any complementary food. In terms of knowledge regarding complementary feeding, 78 % of mothers had knowledge passed down from family, while only 23 % received proper education from their physician [6].

Adequate knowledge and understanding in appropriate feeding practice, development of feeding skills, and establishment of responsive feeding are essential for health workers to be able to detect and manage feeding problems in children. These factors are also required to provide proper education to primary caregivers. Acquiring this knowledge and these skills must be introduced at least in the clinical year of medical education. The existing pediatric nutrition module in our institution does not include comprehensive topics on oral–motor skills in feeding and various feeding behavior. Lectures delivered by a multidisciplinary team of lecturers have also shown changes in student’s knowledge [7]. Clinical teaching by a skill tutorial or simulation (i.e., using simulated patient or media–audio-based clinical simulation) shows improvement in knowledge retention by 90 % compared with 50 % from those who only hear and see [8, 9].

This study aimed to evaluate the effectiveness of a new pediatric nutrition module. This new module is a multidisciplinary educational module that incorporates aspects of feeding from three different specialty areas—Pediatrics, Physical Medicine and Rehabilitation, and Psychiatry—and is delivered using an interactive learning method.

## Methods

This research was a quasi-experimental study. Our inclusion criterion was fifth year medical students who were enrolled in the 2012–2013 academic year in the Medical School, Universitas Indonesia. Any medical students who did not undertake the pre-test and post-test were excluded. Recruitment of subjects was performed by purposive sampling. Written informed consent for participation in the study was obtained from subjects before starting the study.

We considered that a 10-point mean difference in test score between the control and intervention groups would represent an important difference. To detect this difference, with a standard deviation of 10 in the scores, at a 5 % significance level and power of 80 %, we required a minimum of 42 subjects for each group. A pediatric clinical rotation in the Department of Pediatrics takes 9 weeks. There are four rounds of pediatric clinical rotation in 1 year. Therefore, the fifth year medical students were randomly distributed into four groups by the clinical rotation coordinator. In 2012–2013, the groups were as follows: group A (53 students), group B (59 students), group C (50 students), and group D (50 students). As this study required 42 subjects for each group, only two group out of four group which were enrolled for this study. Group assignment for this study was done purposively according to the study time line and pediatric rotation schedule. The control group was a group of students starting pediatric clinical rotation in August 2012 (group C). The intervention module was provided to another group starting pediatric rotation in February 2013 (group A).

The control group was provided the existing module of infant feeding practice, which comprised interactive lectures and a hands-on workshop of case practice papers. This module was delivered by a team of lecturers from the Department of Pediatrics. For the purpose of this study, the existing module is referred to as the control module. The intervention group received a multidisciplinary approach to the infant feeding practice module, which integrated teaching of clinical knowledge and skills from three different departments, the Department of Pediatrics, the Department of Physical Medicine and Rehabilitation, and the Department of Child and Adolescent Psychiatry. Both modules comprised the topics of pediatric nutrition care, infant feeding practice, malnutrition, and weaning problems. The topic of “weaning problems” encompassed an overview of feeding behavior and oral–motor problems, and this topic was delivered by a lecturer from the Department of Pediatrics. However, in the intervention module, these two topics (feeding behavior and oral–motor problems), were addressed in-depth in dedicated lecture sessions. A lecturer from the Department of Physical Medicine and Rehabilitation addressed the topic of “feeding problems associated with oral–motor skill dysfunction” and a lecturer from the Department of Psychiatry addressed the topic of “behavioral approach to infant feeding problems”. A hands-on workshop used the following video-recorded cases were shown in the intervention module.

Two cases were formulated for the essay questions based on common cases that were encountered in daily clinical practice. The first case was a 9-month-old infant who was apparently healthy. Nutritional data were provided as follows. He had rice porridge, which was started when he was 6 months old. He is currently being given blenderized rice porridge composed of rice, vegetables (carrot/spinach), and tofu/fish/chicken, with one small bowl each meal, banana/papaya two times a day, a biscuit once a day, and formula milk five times a day (each 150 mL). The video showed that the mother was feeding the infant in the supine position, exposing the infant to a high risk of food aspiration. The infant was fussy and appeared uncomfortable. He closed his mouth and turned his face away from the spoon. However, the mother was persistent in putting the spoon into his mouth, which made the infant cry during the whole feeding process. The mother did not respond to his crying and still tried to finish the feeding session. The students were asked to analyze the nutritional problem and interaction between mother and infant, arrange pediatric nutritional care, and provide comprehensive education to the mother.

The second case was a 2-year-and-9-month-old girl with good nutritional status. She had three meals a day, snacks on demand, and formula milk two times a day. The video scene showed a lunch session, demonstrating the girl being carried by her mother who walked around the house and the yard. The whole feeding session took 1 h to finish. While feeding her daughter, the mother chatted with several mothers who were also feeding their children and did not pay full attention to her daughter. She also did not respond to her daughter’s cues of feeding refusal, such as turning her face away from the spoon and being cranky. The students were asked to analyze the nutritional problem and interaction between mother and child, to arrange pediatric nutrition care, and provide comprehensive education to the mother.

After watching the video, all of the students were asked to write down their answers and analyses. Discussion for the case was then conducted by a role-play session in which the lecturer acted as a patient. The students played the role of a doctor and were requested to demonstrate medical history taking and provide comprehensive education regarding nutritional problems and infant feeding practice to the patient. The other students were requested to observe and evaluate the role-play session and had an in-depth discussion with the lecturer afterwards. The discussion was then implemented in a role-play session with alternating roles in which the student acted as the patient and the lecturer played the role of the doctor demonstrating good communication and counseling skills. For the purpose of this study, the two modules are referred to as the control and intervention modules. A comparison of both modules is shown in Table 1.

**Table 1:** Comparison of infant feeding practice modules

	Intervention module	Control module
Content	*Department of Pediatrics*	*Department of Pediatrics*
	Pediatric Nutrition Care	Pediatric Nutrition Care
	Infant Feeding Practice	Infant Feeding Practice
	Malnutrition (undernutrition and overnutrition)	Malnutrition
	Weaning Problems	Weaning Problems
	*Department of Child and Adolescent Psychiatry*	
	Behavioural approach to infant feeding problems	
	*Department of Physical Medicine and Rehabilitation*	
	Feeding problems caused by lack of oromotor skills	
Method of workshop	Hands-on workshop using actual cases shown via audiovisual media and role-play session	Hands-on workshop using case practice papers
Lecturer team	Multi-disciplinary team of lectures from Department of Pediatrics, Physical Medicine and Rehabilitation, and Child & Adolescent Psychiatry	A team of lecturers from Department of Pediatrics

Baseline characteristics of the subjects were based on pre-test scores, which may also be perceived as the subjects’ academic performance prior to this particular module. Knowledge comprehension and understanding were assessed based on post-test scores. The pre-test and post-test had an identical set of multiple-choice questions (MCQ) and essays. The pre-test was provided before lectures, while the post-test was provided after the lectures. There were 20 MCQ, which aimed to test the subject’s ability of recalling and analytical skills. The essay questions emphasized infant feeding problems, and focused on evaluating the subject’s skill in problem analysis and implementation of management. This included implementing proper pediatric nutritional care and providing education on appropriate infant feeding practices. Each essay was assessed by two examiners using standardized systematic scoring guidelines. Any score difference of more than 10 % had to be re-assessed and evaluated by both examiners. The scores were then calculated to obtain an average essay score.

As well as the pre-test and post-test, subjects were assigned to take a retention test 3 months following the infant feeding practice module. This test was identical to the pre-test and post-test. An Objective Structured Clinical Examination (OSCE) was administered 6 months after the module and initial testing. There were four stations in the OSCE, which included two stations staffed by the Department of Pediatrics, one station by the Department of Physical Medicine and Rehabilitation, and another station by the Department of Psychiatry. In the pediatrics station, clinical skills of history taking, establishing a diagnosis, comprehensive management, and counseling were assessed. Trigger cases focused on comprehensive management of undernutrition, and failure to thrive attributed to inappropriate infant feeding practices. Physical examinations and identification of problems were the main emphasis of assessment in the medical rehabilitation station. The components being tested involved demonstrating an accurate physical examination and providing education regarding the correct feeding position. In the psychiatry station, subjects were shown a recorded video case of an infant being fed by a caregiver. Students had to assess the interaction between the infant and caregiver at the time of feeding, identify the feeding problem, and provide education as part of management of the feeding problem. Prior to the OSCE, training was provided for OSCE examiners to familiarize them with the scoring panels and minimize any bias or subjectivity in scoring.

Statistical analysis was performed using the *t*-test for data with a normal distribution and the Mann–Whitney test for data with an non-normal distribution. Linear mixed model with random intercept for repeated measures was used to compare pre-test, post-test and retention test scores. Ethical approval for this study was obtained from the Ethical Committee of the Faculty of Medicine, Universitas Indonesia.

## Results

**Fig. 1:**
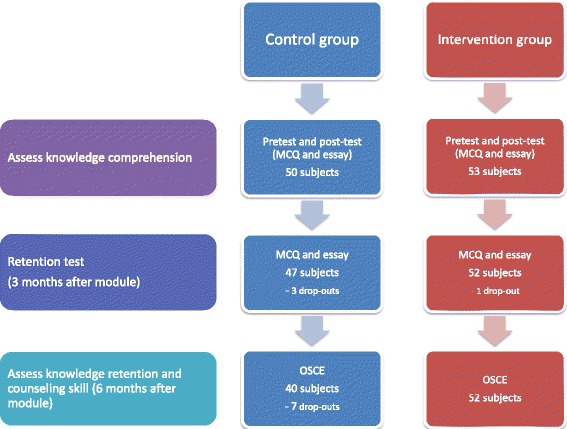
Study subjects’ flowchart

Data collection was performed between August 2012 and March 2013. Initially, there were 50 students assigned to the control group and 53 students assigned to the intervention group. However, in the retention test and OSCE schedule, there were several drop-outs. The study subjects’ flowchart is shown in Fig. 1.

### Subjects’ characteristics

The control group consisted of 50 medical students of whom two students were medical graduates from China undertaking an adaptation program and three students were repeating the pediatric clinical rotation. The intervention group comprised 53 students who were undertaking their pediatric clinical rotation for the first time. Pre-test scores were regarded as baseline scores for general infant feeding practice knowledge. Table 2 shows comparison of pre-test means between the two groups. Pre-test scores in the control group were significantly greater than those in the intervention group (28.4 vs 23.6, *p* = 0.004). However, although statistically significant, in the academic setting, a 4.8-point (score range, 0–100) gap in the score was not considered significant.

**Table 2:** Comparison of pre-test scores between the intervention and control groups

Pretest score	Intervention (*n* = 53)	Control (*n* = 50)	*P*
MCQ score	17.1 ± 4.9	18.8 ± 4.6	0.067
Essay score	6.5 ± 5.6	9.5 ± 5.7	0.007
Total score (MCQ + essay)	23.6 ± 8.1	28.4 ± 8.2	0.004

### Comprehension and understanding of modules

Post-test questions were used to evaluate the students’ comprehension and understanding following the lectures while retention test aimed to evaluate how long the knowledge stays after the modul had been given. Since there were three measurements at three time points (pretest, posttest, and retention test), we performed a linear mixed effect model with a random intercept to analyze to take into account the repeated nature of the data. The mixed effect model demonstrated significant higher result (*p* < 0.001) for pre-test, post-test, and retention test in the intervention group compared to control group as shown in Table 3.

**Table 3:** Result of pre-test, post-test, and retention test using linear mixed effect models

		Group				*P* value
		Intervention (*n* = 52)		Control (*n* = 47)		
		Mean	Standard deviation	Mean	Standard deviation	
Pre-test	Total	23.68	8.07	27.78	7.63	0.001
Post-test	Total	61.65	7.52	57.83	5.68	
Retention test	Total	62.96	10.41	59.21	8.58	

All analyses are comparing pre-test, post-test, and retention test as repeated measurement outcomes. The results in this table were generated from mixed models

### Knowledge retention

The retention test is an assessment tool, which is administered 3 months after lectures and initial testing, to measure retained knowledge. This test consisted of 20-item MCQ and two essay questions, and used the same questions that were used in initial tests (pre-test and post-test). A total of 47 subjects in the control group and 52 subjects in the intervention group undertook the retention test. The post-test scores and retention test scores showed small but significant differences in post-test and retention tests as described in Table 3.

### Evaluation of skill and competence in identification of problems, establishment of diagnosis, and comprehensive management

**Fig. 2:**
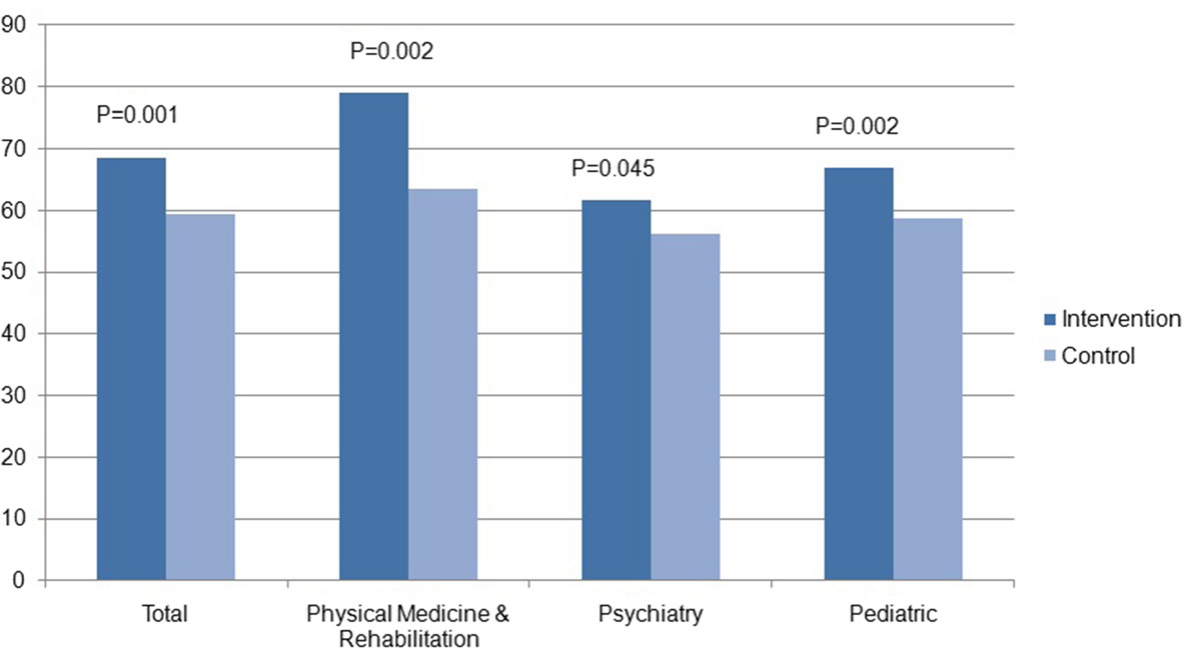
OSCE scores

The OSCE is a research instrument that aims to evaluate subjects’ clinical competence and counseling skills. A total of 40 subjects in the control group and 52 in the intervention group undertook the OSCE. The intervention module yielded better OSCE scores in total means (*p* = 0.001) and in all three study disciplines than those of the control module (Table 4 and Fig. 2).

**Table 4:** OSCE scores

OSCE station	Intervention *n* = 52	Control *n* = 40	Mean difference (95 % CI)	*P*
Pediatrics	67.0 ± 8.7	58.7 ± 10.0	8.3 (3.2;13.3)	0.002^t^
Physical Medicine and Rehabilitation	79.0 (30.0–100.0)	63.5 (20.0–90.0)	15.5 (7.3;23.8)	0.002^mw^
Child & Adolescent Psychiatry	61.6 ± 12.1	56.1 ± 13.5	5.5 (0.1;10.8)	0.045^t^
Total score	68.6 (41.7–84.1)	59.3 (36.5–73.8)	9.7 (5.6;13.9)	0.001^mw^

^t^ *t*-test

^mw^ Mann–Whitney test

### Cost–benefit analysis

There are around 200 students undergoing clerkship in the Department of Pediatrics in a year, divided into 4 rotation, means around 50 students in each clerkship group. The structure of OSCE is holding four sessions of OSCE in a group of clerkship. In every group of clerkship, OSCE test involves 4 examiners and 48 students. Since there are 4 groups per year, the costs for lecture, workshop, and OSCE are multiplied by four in a year. Therefore, the Department will make four OSCE tests and invite, in total, 16 examiners. The OSCE was still a pilot trial performed in this research setting and not yet implemented in the current curriculum. Currently, there is no remedial session for those failed in the OSCE, but in projected simulation, we assume that remedial will be held at the end of year. With the assumption of failure rate in control *vs* intervention is 35.0 % vs 0.58 %, the control group needs four sessions of OSCE tests while the intervention groups only need a session. Cost benefit ratio of the projected scenario will be 2.90 indicating that the cost of the intervention outweighs the benefit (Table 5). The cost effectiveness of the intervention is Rp Rp 130,137 per % of success.

**Table 5:** Cost calculation and cost-benefit ratio

Control module	Unit cost (IDR)	Intervention module	Unit cost (IDR)
Payment for teachers (4 sessions)	3,200,000	Payment for teachers (6 sessions)	4,800,000
Hands-on workshop using case practice papers 2 facilitators	1,600,000	Hands-on workshop using actual cases shown via audiovisual media and role-play session	5,800,000
OSCE (examiners, rooms materials)	8,000,000	OSCE (examiners, rooms materials)	8,000,000
Proportion of students who failed in OSCE	35.0 %	Proportion of students who failed in OSCE	5.80 %
Cost incurred for remedial OSCE, weighted to the proportion of remedial taker	2,666,667	Cost incurred for remedial OSCE, weighted to the proportion of remedial taker	666,667
Cost Benefit Ratio^a^	2.90		
Cost effectiveness ratio 130,137/%success			

^a^CBR = added cost of intervention/benefit of intervention

## Discussion

Our intervention module differs from the control module in terms of integrated comprehensive topics and the delivery method of the module. In the intervention module, two topics were addressed in-depth in dedicated lecture sessions. A lecturer from the Department of Physical Medicine and Rehabilitation addressed the topic of “feeding problems associated with oral–motor skill dysfunction” and a lecturer from the Department of Psychiatry addressed the topic of “behavioral approach to infant feeding problems”. These two topics were discussed in the control module, but not in a dedicated session.

Knowledge of oral–motor skill development is important to determine age-appropriate food. The critical period for training for development of oral–motor skills is at the age of 6–12 months. Therefore, parents should be educated to introduce age-appropriate complementary food during this critical period. Oral–motor delay contributes to feeding problems, which subsequently influence growth and development of children [3, 6].

Another factor that contributes to the success of infant feeding is the parent/caregiver-child interaction in feeding. The feeding style can be divided into four categories of controlling, indulgent, neglectful, and responsive. Controlling parents tend to force their child to eat a certain serving size or restrict meal plans and disregard the child’s wants and needs. In the neglectful style, parents do not have a certain set of boundaries or rules to be applied to the child. Consequently, parents can fail to deliver any of the child’s needs. The indulgent feeding style results when parents excessively respond to a child’s request and encourage having everything he/she wants, regardless of the child’s needs. In contrast, parents who have a clear set of objectives and boundaries sufficiently respond to a child’s needs, and are able to interpret the child’s signals appropriately and demonstrate a responsive feeding style. This feeding style is the most appropriate method and one of the important components in determining successful feeding practice from infancy until the following decades of life [4, 5].

In our study, the intervention module delivery method encouraged active participation and simulation through a hands-on workshop and role-play. The intervention group was shown a video recording of actual cases instead of case practice papers in the control module. The role-play session that was provided in the intervention module was a simulation practice, which aimed to train counseling skill and achieve a higher rate of retention of knowledge. The duration of the hands-on workshop in the intervention module was longer than that in control module, which allowed ample time for comprehensive discussion of each case.

A linear mixed effect model analysis for repeated data (pre-test, post-test and, and retention test) showed higher result for intervention group (*p* < 0.001), indicating that the group receiving the intervention module showed better comprehension of knowledge and understanding. This finding is consistent with our hypothesis that an integrated module and interactive teaching by multidisciplinary staff enhance students’ motivation as well improve the learning process, resulting in better knowledge comprehension. The workshop in the intervention module showed real cases of feeding problems with the aid of audio-visual media. This allowed the students to practice their visual observations and critical analysis skills simultaneously. Knowledge comprehension was evaluated during plenary sessions in the control and intervention groups where students presented their findings. The role-play session, which was only available in the intervention module, allowed greater emphasis on implementation of knowledge, particularly in counselling skills.

The active learning method in the intervention group yielded significantly better results than the control group at the post-test, 3-month post module retention test, and 6-month post-module OSCE, meaning that the intervention group had better understanding, knowledge retention, and counselling skills. As the best method of assessment for competence and practical skills, the OSCE enabled assessment of the students’ performance explicitly by challenging the students to demonstrate how to identify and comprehensively manage the problems based on retained knowledge. Therefore, some aspects of the OSCE promoted retention learning. This result is consistent with Edgar Dale’s cone of experience theory, which states that knowledge comprehension and retention are better with direct application of new knowledge [9]. A previous intervention study without a control showed that the comprehensive learning approach of a nutrition module in medical students yielded better knowledge retention, even at 3 months after the lecture [10].

Our written tests consisted of 20-item MCQ and a two-item essay. The MCQ as an assessment tool is considered as a weak instrument for evaluation of student’s knowledge because students can choose an answer, regardless of proper knowledge comprehension. Our essay enabled extensive evaluation of comprehension of the student’s knowledge. The essay comprised case studies, which required students to identify and analyze problems, determine nutritional status, and provide education to the patient’s caregiver regarding implementation of appropriate feeding practices, including proper feeding behavior.

Evaluation of the essay involved a risk of bias and subjectivity by the examiner. Our study anticipated and minimized such risk by using systematic scoring guidelines, and we had a team of two examiners for examining an essay. Any score difference of more than 10 % required re-evaluation and re-assessment by the designated examiners. This is one of the strength of our study.

**Fig. 3:**
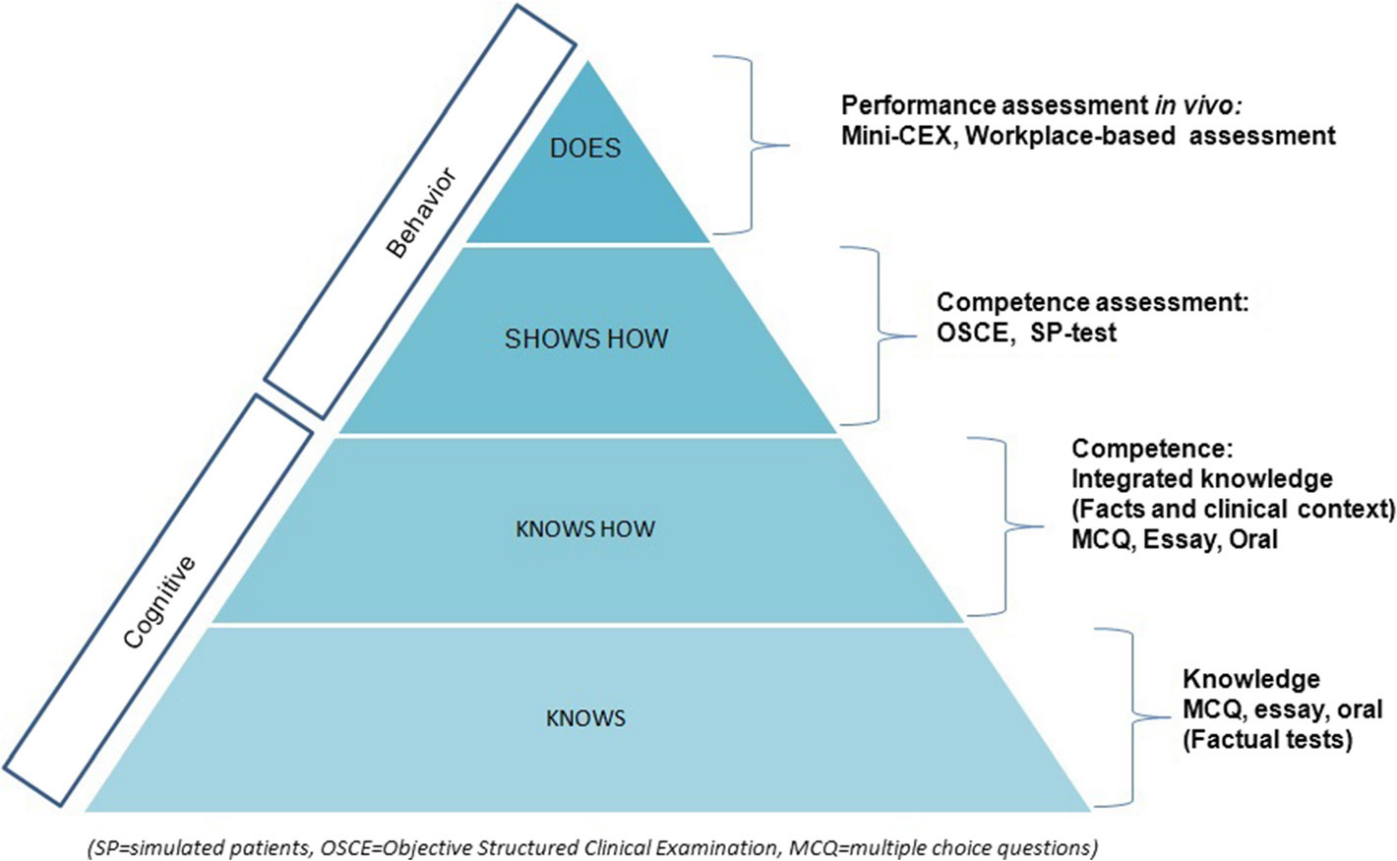
Miller’s pyramid of competency [12]

Assessment of students’ clinical competency can be illustrated by Miller’s pyramid of competence. The base of the pyramid reflects the basic knowledge or is also known as the “know” level. The second tier of the pyramid is called the “know how” level, which involves application of knowledge. These two levels can be assessed using written tests, such as MCQ and problem-solving essays. Accurate assessment of the clinical competence of a doctor requires the ability to show how to implement the knowledge. One of the most appropriate test instruments is the OSCE. The OSCE emphasizes behavioral functioning rather than cognitive function and involves hands-on demonstration of certain skills [11]. During clinical years of medical school, a medical student must be able to demonstrate skills that are required to properly evaluate a patient, and even perform a certain procedure. Ultimately, medical students should possess the knowledge and skills required to ensure that they will become capable and competent doctors in the future. The hierarchy of assessment is shown in Fig. 3.

In the current study, the retention test’s score in the intervention group was significantly higher than that in the control group. This finding, along with significantly higher OSCE scores, further supports that the intervention module delivered a better learning processes and yielded better retention along with improved practical skills of medical students.

A limitation of our study was that random allocation to the control and intervention groups could not be performed because of clinical rotation scheduling restraints. Consequently, some factors affecting unequal distribution of the subject population could not be avoided. One of the most contributing factors in this case was the academic performance of each student. Our study also lacked evaluation of students’ perspectives and feedback regarding the effectiveness of the intervention module.

With regard to assessment methods in an educational setting, using the OSCE as our research instrument to evaluate students’ knowledge and practical skills was one of the strengths of this study. The students were also assessed using a combination of MCQ and essays to yield more information of the students’ performance in relation to knowledge comprehension. Examiners’ bias from essay scoring was minimized by having two examiners for each essay and using a point-by-point guideline for answer scoring.

The findings of our study would be useful for further developing and improving the current pediatric nutritional care curriculum that is administered to fifth year medical students in the pediatric clinical clerkship program. Incorporating integrated learning processes by multidisciplinary teaching staff, interactive hands-on workshops involving learning cases with the aid of audio-visual media, and a role-play session were effective in our study for improving knowledge comprehension and retention, especially for acquiring applied skills required to produce competent and skilled future doctors.

The cost–benefit analysis was not a primary outcome of this study. This study was not designed to evaluate the final benefit in terms of a reduction in health expenses as an indirect effect of decreased infant and child undernutrition due to improved knowledge and skill of doctors. However, implementation of the intervention module requires additional multidisciplinary staff, which involves staff from the Departments of Pediatrics, Physical Medicine and Rehabilitation, and Child and Adolescent Psychiatry. This implementation will also lead to additional costs for lecturers and administrative officers, development of teaching materials, OSCE arrangements, and room allocation. Therefore, we attempted to calculate the cost–benefit ratio, which views the benefit in terms of the percentage of students who passed the OSCE. Cost–benefit ration of the projected scenario was 2.90, indicating that the cost of intervention outweighs the benefit. However, despite the cost, OSCE is the most feasible and reliable method to evaluate the competence as practicing physician. Thus, OSCE should still be considered to be a tool to measure knowledge and skill of the medical undergraduate student.

The feasibility of fulfilling certain needs to implement the intervention module must be discussed and evaluated by the University’s general board and education committee. The effectiveness of the intervention module, in terms of knowledge comprehension and retention, needs to be re-assessed and evaluated after the students have graduated from medical school and started professional practice. Workplace-based assessment, which reviews the students’ actual performance during clinical practice, is considered the best method to evaluate their competence as practicing physicians.

## Conclusions

Acquiring knowledge of infant feeding practice and its counseling skill in the early period of medical education, ideally prior to clinical clerkship, is of paramount importance. Multidisciplinary medical education with an interactive learning method and clinical teaching with simulation, such as role-play and audio-visual-based case simulation, improve knowledge comprehension and retention of undergraduate medical students. Implementation of such an essential module of infant feeding practice along with an effective teaching method are pivotal to ensure that doctors of future generations are equipped with the necessary knowledge and skills, and thus become competent. Ultimately, they are prepared to overcome infant and child mortality related to feeding problems.

## Abbreviations

MCQ, multiple-choice questions; OSCE, Observed Structured Clinical Examination
